# Noninflammatory Causes of Pulmonary Edema During Ex Vivo Lung Perfusion

**DOI:** 10.1016/j.atssr.2024.06.013

**Published:** 2024-06-28

**Authors:** Jennifer Whiteley, Hongchao Shan, Jonathan C. Yeung

**Affiliations:** 1 Toronto Lung Transplant Program, Toronto General Hospital, Toronto, Canada

## Abstract

Ex vivo lung perfusion (EVLP) is used to evaluate donor lungs prior to lung transplantation. Development of pulmonary edema during EVLP is generally thought to represent inflammatory breakdown of the air-fluid barrier and these lungs are declined for transplant. We present the case of a donor lung that underwent stapled wedge resection during cold storage for air leak and the subsequent development of profound (∼650 mL) pulmonary edema around the staple line during EVLP. Nevertheless, the edema cleared shortly after implantation. This report illustrates the potential for significant alveolar fluid clearance and sealing of vascular injury after implantation when edema is not caused by inflammatory injury.

Acellular ex vivo lung perfusion (EVLP) offers the ability to assess donor lungs before transplantation; however, acellular lung physiology affects the interpretation of Po_2._^1^ Instead, the current EVLP evaluation strategy examines surrogates of pulmonary edema development over time. These include perfusate loss, lung weight, chest radiography, metabolites, and physiologic parameters such as compliance and airway pressure.^2^^,^^3^ Pulmonary edema formation during EVLP is thought to represent the inflammatory (ie, acute respiratory distress syndrome-like) microvasculature injury caused by pneumonia, aspiration, or reperfusion injury which would have resulted in primary graft dysfunction following implantation into the recipient. Therefore, edematous lungs during EVLP are declined for transplant.

One additional consideration is that the acellular perfusate used in EVLP lacks clotting factors to plug physical damage to the vasculature. Thus, not all EVLP edema may be due to inflammatory injury. Anecdotally, some lungs become slightly edematous during EVLP, but dry out rapidly after implantation into the recipient. We hypothesized that noninflammatory breakdown of the microvasculature such as from volume overload or mechanical injury may cause edema during EVLP, but these lungs may still be usable, as implantation into the recipient will allow for platelet plugging of the injury and physiologic alveolar fluid clearance mechanisms are preserved.

Herein, we present a case of a pure mechanical injury to the donor lung during EVLP, the resultant profound edema formation during EVLP, and demonstration that this edema can rapidly resolve after implantation into the recipient.

The lung transplant recipient provided consent for publication.

A 62-year-old male patient offered donation after cardiac death and had a P/F ratio of 596 mmHg. The donor arrested at 181 minutes after withdrawal of life-sustaining therapies. Procurement was uncomplicated but an apical bulla at the apex of the right lung with air leak was stapled to prevent deflation after procurement and during cold storage.

Due to the long agonal phase, we elected to assess the lungs on EVLP.

After 1 hour of EVLP, there was significant perfusate loss (650 mL) into the lung. The right upper lobe became profoundly heavy and edematous. Right superior venous gas Po_2_ was 30 mm Hg and outflow venous Po_2_ was 369 mm Hg. Bronchoscopy showed edema fluid from the right upper airway. The EVLP lung radiograph showed dense infiltrates limited to the right upper lobe (Figure 1). We felt that the edema was due to perfusate leak from the staple line into the lung and terminated EVLP prematurely at 1:50 hours.

**Figure 1:**
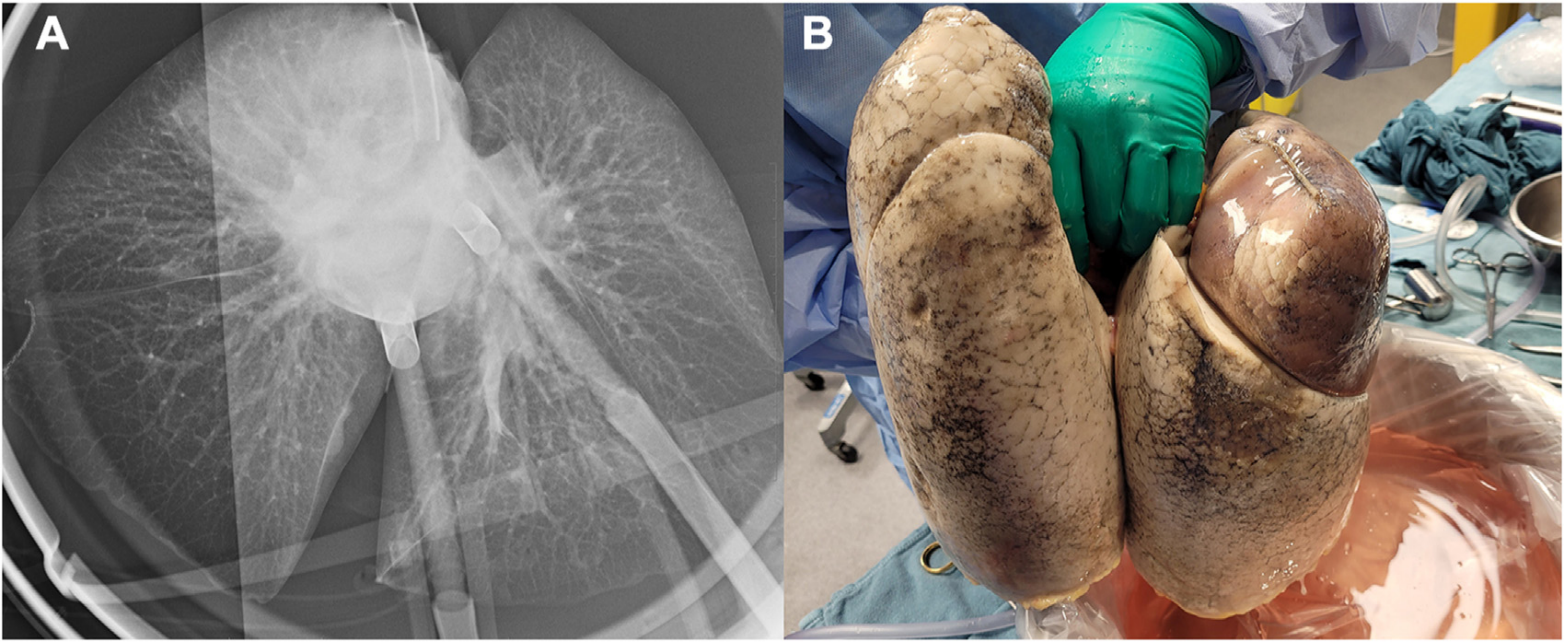
(A) Radiograph of ex vivo lung showing opacity in the right upper lobe. (B) Photo of donor lungs after ex vivo lung perfusion.

The recipient was a 68-year-old male individual with end-stage chronic obstructive pulmonary disease. Intraoperative central venoarterial extracorporeal membrane oxygenation (VA ECMO) was used to reduce perfusion to the transplanted lung and the edematous right lung was transplanted first to allow for alveolar fluid clearance. Suctioning of the right upper lobe bronchus was performed prior to implantation to remove edema fluid. The transplant was otherwise uncomplicated and the first postoperative P/F ratio was 480 mm Hg. The chest radiograph upon arrival to the intensive care unit showed a clear right upper lobe (Figure 2). He remains well 1 year after transplant.

**Figure 2:**
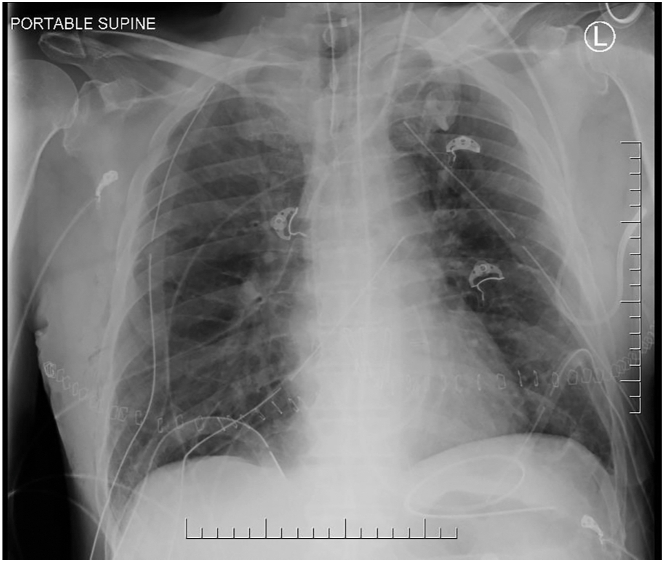
Chest radiograph of recipient upon arrival to the intensive care unit.

## Comment

In this case report, we highlight an alternative cause of EVLP pulmonary edema caused by noninflammatory injury during EVLP and the alveolar fluid clearance that can occur in this circumstance.

Stapling of the lung is a common occurrence in lung surgery; however, for this case, the stapling was performed during cold storage without opportunity for platelet plugging and initiation of inflammation. The lung was then perfused with an acellular perfusate, again without opportunity for platelet plugging and inflammation, leading to the development of profound pulmonary edema in the right upper lobe. Despite this, we show that these lungs could be utilized safely for transplantation and that there is rapid clearance of pulmonary edema (almost 650 mL) after implantation.

This case report advances the hypothesis that pulmonary edema during EVLP caused by noninflammatory causes may be safely transplanted due to preserved alveolar fluid clearance mechanisms. For inflammatory lung injury, studies in acute respiratory distress syndrome have demonstrated that the breakdown in the microvascular barrier is accompanied by a reduced ability for lungs to clear alveolar edema fluid.^4^ However, in lungs without inflammatory microvascular injury, alveolar fluid clearance can be rapid; in a pig model, there was 50% clearance of alveolar fluid by 4 hours.^5^ Along those lines, we chose to implant the edematous lung first on VA ECMO to reduce hydrostatic pressure in the pulmonary vasculature and to provide time for alveolar fluid clearance mechanisms to work during implantation of the second lung. Indeed, by the time of separation from VA ECMO, the upper lobe was much less edematous and the chest radiograph taken on arrival to the intensive care unit showed a clear right upper lung field and overall excellent lung function and long-term outcome.

EVLP lung evaluation requires clinical judgment and expertise in the physiology of lung injury. Identifying causes of noninflammatory lung injury such as donor volume overload and physical injuries may yet allow for more lungs to be safely transplanted off EVLP.
